# Human Myometrial Cell Fate under Chronic Oxidative Stress for Leiomyomagenesis

**DOI:** 10.21203/rs.3.rs-9323630/v1

**Published:** 2026-07-20

**Authors:** Jianjun Wei, Matthew Schipma, Yue Feng, Wenan Qiang, David Gius, J. Julie Kim

**Affiliations:** Northwestern University; Northwestern University; Northwestern University; Northwestern University; Northwestern University; Northwestern University

**Keywords:** Myometrium, Oxidative stress, MnSOD, Transcriptome, Leiomyoma, MED12

## Abstract

Uterine Leiomyoma are the most common benign tumors in women of reproductive age. Studies show that both leiomyoma and surrounding myometrium exist in a state of high oxidative stress, characterized by reactive oxygen species (ROS) production and impaired antioxidant defenses including defects of manganese superoxide dismutase (MnSOD). Oxidative stress has been proposed as a driver of leiomyoma initiation by promoting oxidative DNA damage leading to mutations in MED12 and leiomyoma growth. We hypothesize that chronic oxidative stress in human myometrial cells induces cellular remodeling and genomic alterations that promote adaptation and contribute to early events in leiomyoma development. To test this hypothesis, we generated stable human myometrial cell lines (Myo-hTERT) of wild type, MnSOD^68K^ and mutant MnSOD^68Q^ that impairs MnSOD antioxidant activity thereby imposing sustained oxidative stress. Experiments were performed both in vitro using three-dimensional spheroid cultures and in vivo using xenograft models in immunocompromised mice, with some studies extending up to 10 months to model chronic oxidative stress. Cells were exposed to the oxidative inducer paraquat (PQ) and analyzed using cellular and molecular approaches including spatial transcriptomics and *MED12* mutational analysis. Cells expressing MnSOD^68Q^ exhibited increased intracellular ROS and oxidative DNA damage, as indicated by accumulation of 8-hydroxy-deoxyguanosine (8-OHdG). Transcriptomic analysis revealed activation of pathways associated with oxidative stress responses including collagen/extracellular matrix, cellular senescence and IGF/AKT signaling. Xenografts of primary myometrial cells subjected to prolonged oxidative stress demonstrated similar molecular responses and showed an increased frequency of *MED12* mutations. These changes were accompanied by modest increases in cell proliferation with minimal induction of cell death. Together, these findings provide mechanistic evidence that sustained oxidative stress can drive molecular and genomic changes in myometrial cells and support a causal role for chronic redox imbalance in the initiation of uterine leiomyomas.

## Introduction

Uterine leiomyomas are benign smooth muscle tumors that affect up to 70% of women of reproductive age^1^. While their exact etiology remains unclear, increasing evidence implicates oxidative stress, which is an imbalance between ROS production and antioxidant defenses in myometrial cells, as a critical contributor to leiomyoma development and growth. Oxidative stress promotes DNA damage through the formation of oxidized nucleotides such as 8-oxoguanine (8-OHdG), which can lead to mutagenic misrepair^2^. In particular, oxidative DNA damage has been proposed to contribute to somatic mutations in *MED12*, including the recurrent double G at c130–131 in *MED12* exon 2 that are hallmarks of uterine leiomyomas^3,4^. We previously demonstrated that high levels of oxidative exposure *in vitro* induce oxidized nucleotide accumulation and DNA damage in myometrial cells supporting a link between ROS-mediated genomic instability and leiomyoma initiation^4^.

While low and moderate ROS levels facilitate normal cellular signaling and proliferation^5^, excessive ROS can damage DNA, disrupt mitochondrial function, and promote tumorigenic processes^6^. Uterus is a hormonally active tissue that experiences substantial oxidative stress, and myometrial cells thus reside in a relatively oxidative environment^7^. Persistent ROS production could lead to mitochondrial dysfunction and promote a pro-inflammatory microenvironment characterized by increased cytokine signaling and ROS generation further amplifying oxidative burden^6^. In addition, estrogen and progesterone, key drivers of leiomyoma growth, can indirectly enhance ROS production by stimulating NOX activity and mitochondrial respiration^8^. Clinical and histologic evaluation demonstrate that leiomyoma tissues exhibit lower MnSOD activity compared to adjacent normal myometrium, accompanied by elevated levels of the oxidative DNA damage marker, 8-OHdG^9^.

MnSOD, encoded by *SOD2*, is a nuclear-encoded mitochondrial antioxidant enzyme that serves as a primary defense against mitochondrial oxidative stress. MnSOD catalyzes the conversion of superoxide radicals (O_2_^−^), generated during mitochondrial respiration, into hydrogen peroxide (H_2_O_2_) and molecular oxygen (O_2_), thereby preventing the accumulation of highly reactive superoxide species^10^. MnSOD activity is tightly regulated by genetic, epigenetic, and hormonal mechanisms, and disruption of this regulation can compromise mitochondrial redox balance. In leiomyoma cells, epigenetic alterations such as hypermethylation of the *SOD2* promoter or histone deacetylation have been reported to suppress MnSOD expression, leading to increased mitochondrial ROS leakage and amplification of oxidative stress^11^. Acetylation of MnSOD at lysine 68 (K68) neutralizes the residue’s positive charge and alters the enzyme’s catalytic properties^12^. This modification destabilizes the MnSOD tetramer—the configuration required for optimal superoxide dismutase activity—thereby reducing its capacity to detoxify mitochondrial superoxide. Instead, acetylated MnSOD favors monomeric or dimeric forms that exhibit peroxidase-like activity, which can promote the accumulation of mitochondrial reactive oxygen species and link mitochondrial metabolism to disease progression^13^. The MnSOD-K68Q mutant, widely used as an acetylation mimic, models constitutive K68 acetylation by neutralizing the lysine charge and impairing canonical MnSOD dismutase activity. Accumulation of MnSOD-AcK68 has been associated with altered mitochondrial structure, metabolic reprogramming, and resistance to cancer therapies, contributing to a pro-tumorigenic phenotype^14^.

In this study, we sought to determine whether sustained oxidative stress is sufficient to alter myometrial cell fate and contribute to early events associated with leiomyoma initiation. Building on our prior observations of elevated ROS and impaired MnSOD activity in leiomyoma, we engineered myometrial cells to express an acetylation-mimic MnSOD mutant that reduces superoxide detoxification capacity, thereby imposing chronic oxidative stress. Using engineered myometrial cell lines, primary human myometrial cells and complementary in vitro and in vivo models, including long-term studies extending up to 10 months, we examined how persistent ROS exposure promotes genomic alterations and activates molecular pathways characteristic of uterine leiomyomas that contribute to leiomyoma initiation. These experiments directly test whether prolonged redox imbalance can drive cellular changes rather than simply correlate with leiomyoma development, providing mechanistic evidence that chronic oxidative stress may contribute to the earliest stages of leiomyoma formation.

## MATERIALS AND METHODS

### 1. Materials, cell line, SOD constructs, stable cell establish

Myo-hTERT cell line was kindly provided by C. Mendelson (UT Southwestern cultured in DMEM/F12 Medium (Thermo Fisher Scientific, Cat# 41966052) plus 10% Fetal Bovine Serum (Fisher Scientific). Primary myometrial cells were isolated as previously described^15^. All the cells were grown at 37°C in a humidified cell culture incubator containing 5% CO2. Media was changed every 48 h, and cells were passaged at 90% confluence.

MnSOD lysine 68 variant constructs of wild type (68K) and mutant MnSOD^AC68Q^ (68Q, acetylated) were kindly provided by Dr. David Gius. Lentivirus infected myo-hTERT with stable expression of wild type and mutant MnSOD were established (cisplatin and doxorubicin resistant myo-hTERT cells were treated for over 3 months to establish permanent cell lines^12^. Briefly, human Lenti-MnSOD plasmid was used for site-directed mutagenesis where lysine at location 68 is mutated to glutamine (acetylation mimetic) as previously described^12^. HEK 293T cells were transfected with 5μg of the target DNA, 5μg of the psPAX2 packaging plasmid, and 300 ng of the VSV-G envelope plasmid. Fresh medium was added after overnight incubation; the viral supernatant was collected 48 hours later and filtered using a 0.45 μm filter (Corning). When Myo-hTERT cells reached 40% confluence, viral infection was performed in the presence of 10 μg/ml Polybrene for 72 hours. Subsequently, the cells were allowed to recover in culture medium for 24 hours and were then selected using 1 μg/ml puromycin. For each genotype, at least five monoclonal colonies were picked and verified by DNA sequencing analysis (**Suppl Fig. S1A**). All experiments were done using exponentially growing cells at 50%−70% confluence.

### 2. Cell culture

Oxidative stress conditions were mimicked in vitro by the exogenous addition of Paraquat dichloride hydrate (PQ; Sigma Aldrich, 36541). An optimal dose for PQ (100 μM) was selected based on ~ 50% cell death after 48 hrs. of treatment in a dose-response study. Human myometrial and leiomyoma tissue samples were from patients with informed consent. All experiments were carried out in accordance with the guidelines and regulations. The study was reviewed and approved by the Northwestern University Institutional Review Board (IRB) Office with IRB approval number: STU00018080. Primary cultures were obtained from myometrial tissues as previously described with minor modifications^16^. Briefly, the tissue was minced into small pieces and washed twice with cold Dulbecco’s phosphate-buffered saline (DPBS; Life Technologies) containing 1% antibiotic-antimycotic solution; subsequently, it was digested for 5 hours at 37°C in Hank’s balanced salt solution (HBSS; Life Technologies) containing 1.5 mg/mL collagenase (Sigma-Aldrich, St. Louis, MO), 83.3 μg/mL DNase I (Sigma-Aldrich), and 1% antibiotic-antimycotic solution. The cell suspension was filtered through a 100 μm nylon cell strainer (BD Falcon, Bedford, MA). Cells (5–10 × 10^6^) were seeded into 100 mm culture dishes and cultured in smooth muscle cell culture medium supplemented with 1% antibiotic-antimycotic solution. All cells were cultured in a humidified incubator at 37°C with 5% CO_2_.

### 3. Spheroid/three-dimensional (3D) culture

Myometrial primary and cell lines spheroids were cultured as previously described^15,17^. Briefly, cells were seeded into 96-well ultra-low attachment culture plates (Corning Costar). The cells were cultured in mesenchymal stem cell medium (Lonza, Cat. No. PT-3001) and incubated in a humidified incubator at 37°C with 5% CO2 for at least 48 hours. Subsequently, the formed cell spheroids were observed and evaluated.

### 4. Western blot with crosslink

Cells were washed three times with 1x PBS, harvested, and lysed for 30 min in radioimmuno-precipitation assay (RIPA) buffer (Thermo scientific TC263138) with Halt^™^ Protease and Phosphatase Inhibitor Cocktail (Fisher Scientific 78440), then quantified by Pierce BCA protein assay and immunoblotted with: anti-MnSOD (1:1000 dilution, Millipore, #06–984) Incubate with gentle rocking overnight at 4°C, Secondary anti-rabbit (1:5,000 dilution, Cell Signaling, #7074). For the MnSOD tetramerization assay for crosslink, lysed cells were treated with 0.1% glutaraldehyde for 10 min at room temperature before samples were immunoblotted with anti-MnSOD antibody. Primary antibodies included β-tubulin (Proteintech, 1;10,000 dilution), GAPDH (Proteintech, 1:10.000 dilution), MnSOD (Millipore, 1;1,000 dilution), MnSOD-K68-Ac (Abcam, 1;1,000 dilution). The original Western Blot film was presented in Supplementary Figure S5.

### 5. ROS evaluation: DHE, MiTSOX

Cells were washed with cold PBS, immediately fixed with cold methanol (−20°C) for 10 minutes, and subsequently incubated in PBS containing 3% bovine serum albumin (BSA) for 1 hour to block non-specific binding sites. Primary antibodies against 8-OHdG (Santa Cruz) or γH2AX (Novus Biologicals) were added, and the samples were incubated overnight at 4°C. After washing three times with 1X PBST, the secondary antibody—Alexa Fluor^®^ 488-conjugated goat anti-mouse antibody (1:1000, Life Technologies)—was added, and the samples were incubated for 1 hour at room temperature. Slides were counterstained with 4′,6-diamidino-2-phenylindole (DAPI) for analysis. For DHE staining, slides were incubated with 5 μM DHE on an orbital shaker at room temperature for 10 to 15 minutes, protected from light. Slides were washed three times with 1X PBS and lightly fixed in 1X PBS containing 7% formaldehyde for 4 to 8 minutes; subsequently, they were counterstained with DAPI, and images were acquired using a fluorescence microscope.

MitoSOX^™^ Green (MSG) and Red (MSG) reagent working solutions were prepared based on protocols provided by vendor (Invitrogen). 1–2 mL of the MSR reagent or MSG reagent working solution was applied to cover cells adhering to coverslip(s) in a well of 35 mm dish, or 100 μL per well of 96 well plate. Cells were incubated for 30 minutes at 37°C and 5% CO2 under the dard field. Cells were washed gently 3 times with warm buffer (HBSS with Calcium and Magnesium or suitable buffer). Cells will be reviewed using spectral properties within 2 hours of staining (detail see instruction by vendor).

### 6. Immunohistochemistry

immunohistochemical (IHC) was performed on a Ventana Nexus automated system, as described previously^9^ in formalin-fixed and paraffin-embedded tissue, including H01, MnSOD, Ki67, ER. Intensity was scored as negative (0), weak (1+), moderate (2+), or strong (3+) and percentage of positive tumor cells was scored from 0% to 100%.

### 7. Tissue microarray

Following sectioning and microscopic review, FFPE tissue samples were selected from subcutaneous xenograft (cell line) and subrenal capsule graft (primary myometrial tissue) nodules. Subsequently, 1.0 mm diameter tissue cores were extracted to construct tissue microarrays (TMAs), which were then sectioned at a thickness of 4 μm. To ensure quality and verify the accuracy of the tissue types, hematoxylin and eosin (H&E) staining was performed on the first and last sections of each TMA.

### 8. RT-PCR

Total RNA was extracted from fresh cells using TRIZOL reagent (Invitrogen, Carlsbad, CA) and from formalin-fixed, paraffin-embedded (FFPET) tumor tissues using FFPET RNA Isolation Kit according to the manufacturer’s (Qiagen) instructions. RNA concentration was measured using a NanoDrop (ND-1000, Saarbrücken). cDNA was synthesized using the PrimerScript RT Reagent Kit (TaKaRa). Quantitative RT-PCR (qRT-PCR) was performed using SYBR Green PCR Master Mix on an Applied Biosystems 7900HT real-time PCR system. mRNA expression levels in each sample were normalized to the internal control gene GAPDH. All experiments were performed in triplicate.

### 9. RNA-seq

Total RNA was extracted using the Qiagen miRNeasy Mini Kit according to the manufacturer’s instructions (Qiagen, Cat. No. 217004). RNA samples were quantified by a Qubit 2.0 fluorometer (Life Technologies), and RNA integrity was assessed by an Agilent TapeStation 4200 (Agilent Technologies). RNA libraries were constructed with RNA Integrity Number (RIN) of 9 or higher using the NEBNext Ultra II RNA Library Prep Kit for Illumina, following the manufacturer’s instructions (New England Biolabs/NEB, Ipswich, MA) as previously described^4^. Briefly, mRNAs were enriched using Oligo (dT) beads. First strand and second strand cDNA fragments were end repaired and adenylated at the 3’ ends. Universal adapters were ligated to the cDNA fragments, followed by the addition of indices and library enrichment via limited-cycle PCR. Quality control cDNAs were prepared using an Agilent TapeStation (Agilent Technologies), and quantified by Qubit 2.0 fluorometer (Invitrogen) or quantitative PCR (KAPA Biosystems). RNA sequencing was performed by Genewiz (Genewiz, NJ) on an Illumina HiSeq 4000 platform using a 2×150 bp paired-end (PE) sequencing mode.

### 10. RNA sequencing processing and analysis

The quality of DNA sequencing reads in FASTQ format was assessed using FastQC (Babraham Bioinformatics). Adapters were trimmed, and low-quality reads or reads mapping to rRNA sequences were removed. Cleaned reads were aligned to the human GRCh38 reference genome (from the ENSEMBL database) using the STAR aligner (v.2.5.2b), and unique gene hit counts were calculated using the featureCounts tool from the Subread package (v.1.5.2). Following the extraction of gene hit counts, differential expression analysis was performed using the DESeq2 package in R. Genes were identified as differentially expressed based on established criteria: an adjusted p-value < 0.05 and an absolute log2 fold change > 0.5 (for the Control vs. PQ comparison) or > 1 (for the Control vs. KBrO3 comparison)^4^. To perform cluster analysis on a set of samples, all genes and their corresponding RPKM expression values within the group were first aggregated to construct a read count matrix. Subsequently, this integrated read count matrix was analyzed using unsupervised learning and other machine learning techniques. The R packages ggplot2 and Pheatmap were employed to generate heatmaps and volcano plots, while Factoextra and FactoMineR were used to conduct principal component analysis (PCA), revealing similarities between samples based on distance matrices. The “fgsea” and “enrichplot” packages were run for gene ontology (GO) and Kyoto Encyclopedia of Genes and Genomes (KEGG) analysis (ShinyGO 0.85 https://bioinformatics.sdstate.edu/go/^18–20^. Functional analysis was performed by Gene Set Enrichment Analysis (GSEA) using the Molecular Signatures Database, MSigDB (http://www.broad.mit.edu/gsea/msigdb/index.jsp).

### 11. DNA sequencing for MED12 mutation

DNA was extracted from fresh/frozen or FFPE tissues using the Quick-DNA^™^ MiniPrep Plus Kit and Quick-DNA^™^ FFPE Kit (Zymo Research, USA) according to the manufacturer’s protocols. For Sanger sequencing, 50 ng of genomic DNA was used to amplify exon 2 of the MED12 gene with the following primer sequences: 5’-ACAACTAAACGCCGCTTTCCT-3’ (forward) and 5’-GGGCCTTTGCTCCTTCTTAG-3’ (reverse) as previously described^4^. Sanger DNA sequencing of purified DNA products was performed in NUSeq Core (Northwestern) with Applied Biosystem’s 3730xl DNA Analyzer. Mutations/variations were analyzed by FinchTV and Indigo software (https://www.gear-genomics.com/indigo/).

### 12. Xenograft in mice

All animal experiments and methods were performed in accordance with the guidelines and regulations with an approval of Northwestern University Animal Care and Use Committee (ISOOO15081). Female NOD.Cg-Prkdcscid Il2rgtm1Wjl/SzJ (NOD) mice (aged 6–8 weeks) were injected subcutaneously in bilateral flanks with cell suspensions (5×10^6^ cells in 100 μl PBS) mixed with Matrigel. A total of 12 wild types (12x MnSOD^68K^) and 12 mutant types (12x MnSOD^68Q^) xenografts were prepared. All conditions were split into controls and treated groups, 6 mice each. Mice from nontreated groups provided routine food and water and treated groups provided water with Paraquat dichloride hydrate (PQ, Sigma 36541) in dosage of 10mg/kg/day in water.

The xenografting of primary myometrial cell pellets has been described previously^16^. Myometrial cells were suspended in rat tail collagen (type I) solution (BD Biosciences, San Jose, CA) at a density of 5–10 × 10^5^ cells per 20 μl. The cell suspension was seeded and incubated at 37°C for 30 minutes to allow collagen gel polymerization^21^, followed by overnight culture in DMEM/F-12 medium supplemented with 10% fetal bovine serum (FBS) and 1% antibiotic-antimycotic solution. The cell aggregates were transplanted under the kidney capsule of 6–8-week-old NOD-scid (NSG) mice (Jackson Laboratory, Bar Harbor, ME); the recipient mice had undergone ovariectomy (OVX) and subcutaneous implantation of slow-release E2 + P4 hormone pellets (composition: 0.8 mg E2, 75.2 mg P4, and 5 mg cholesterol; total weight: 80 mg).

For Ovariectomy or SubQ implantation of hormone pellets, vaporizors (VetEquip) rent were used and set 2–5% for induction and 1–3% for maintenance by Inhalation. For Renal capsule grafting,100mg/kg Ketamine and 10mg/kg Xylazine mixture were administrated by intraperitoneal. The depth of anesthesia will be monitored by respiration status, reflex to pinching of toe and foot pad. Additional dosing of ketamine (2 mg/kg) was given if the mouse was not completely anesthetized 5 minutes after the second injection. Animals recovered from anesthesia by placed in a cage on a warming pad as previously described^22^.

Mice euthanasia was performed at 12 weeks for renal capsule xenografts and 40 weeks for subcutaneous implantation. Mice were euthanized by gradual-fill CO2 inhalation in accordance with our IACUC guidelines. Following cessation of respiration, a secondary physical method (cervical dislocation) was performed to ensure death. Tumor volume was calculated according to the formula TV (cm3) = a × b2 × π/6, where ‘a’ is the longest diameter, and ‘b’ is the shortest diameter. H&E and immunostains were performed on sections from embedded tissue samples as previously described^22^.

### 13. Spatial transcriptome of single cell RNA-Sequencing

Detail method has been described in our previous study^23^. In brief, A tissue microarray (TMA) containing a 4×4 grid of 1 mm tissue cores within a 6×6 mm area was prepared for spatial transcriptomic RNA sequencing. Concurrently, regions of interest were identified through morphological examination of H&E-stained sections. To assess RNA quality, 1–2 sections (5 μm thick each) were obtained from each FFPE tissue block, and RNA was extracted using the FFPE kit (Qiagen 73504) according to the manufacturer’s instructions. The extracted RNA was analyzed using the Agilent Bioanalyzer with an RNA Pico chip to confirm a DV200 value greater than 30%.

For each sample, a 5 μm-thick section was placed on a Visium slide and incubated at 42°C for 3 hours, followed by overnight incubation at room temperature. Subsequently, the slides were deparaffinized for H&E staining. Decrosslinking was performed according to the protocol established by 10x Genomics for the Visium HD Spatial Gene Expression kit (10x Genomics, 1000673). Next, human probe (v2) hybridization and ligation reactions were conducted on the sections using the 10x Genomics Visium HD Spatial Gene Expression (Human, 6.5 mm) kit (10x Genomics, 1000673). The CytAssist instrument was used to release the probes from the tissue sections and capture them onto the Visium slide, followed by probe extension. Sequencing libraries were then prepared. Pooled libraries were sequenced on a NovaSeq X Plus sequencer using a 100-cycle kit, with parameters set to 50 nt for Read 1 and 50 nt for Read 2. The visium slide processing, library preparation and sequencing was done at Northwestern University NUSeq facility core. The datasets with a title of ‘Human Myometrial Cell Fate’ generated and/or analyzed during the current study are available in the NCBI Gene Expression Omnibus (GEO) repository (https://urldefense.com/v3/__https://www.ncbi.nlm.nih.gov/geo/query/acc.cgi?acc=GSE330163__;!!Dq0X2DkFhyF93HkjWTBQKhk!X7hrshV01rvYutT6c7Cz41atKOVS-RI-WBoHYXdpIFnQQPQYcCrxjusAfV_qqqcXJwxcFdrSLPcA2oAcgLAlj1Qotno$) with accession number: GEO330163.

### 14. Spatial Transcriptomics Analysis

Detail method has been described in our previous study^23^. In brief, Raw sequencing data were demultiplexed and converted to FASTQ format using BCLConvert (version 4.3.6) on the NovaSeq X Plus sequencer. Images were loaded and manually registered using Loupe Browser (version 8.1.2). Space Ranger (version 3.1.2) was used to align FASTQ files against the human probe set and images, quantifying the number of reads mapped to each probe per cell. The resulting matrices were imported into Seurat (developed by the Satija Lab at NYGC) for downstream analysis. Within Seurat, each sample underwent preprocessing, normalization, and scaling. Subsequently, the *Integrate Data* function in Seurat was used to merge all samples into a single dataset, and metadata containing original sample information was added. All UMAP plots, violin plots, spatial feature expression plots, and heatmaps were generated using Seurat tools. Cell type annotation was automatically performed using the scType R package.

### 15. Statistics

Statistical analysis of RNAseq was performed using related packages in R. GraphPad Prism version 8.0 (GraphPad Software) was used for ordinary paired t-test, one-way ANOVA, Brown-Forsythe or Kruskal-Wallis test when performing for multiple comparisons depending on the distribution and variances of the data. All data represent the mean ± SEM of a minimum of three independent experiments and data was considered statistically significant if the p value was < 0.05.

## RESULTS

### Acetylation-mimics of MnSOD at K68 Enhances Oxidative Stress in Myometrial Cells

**Figure 1:**
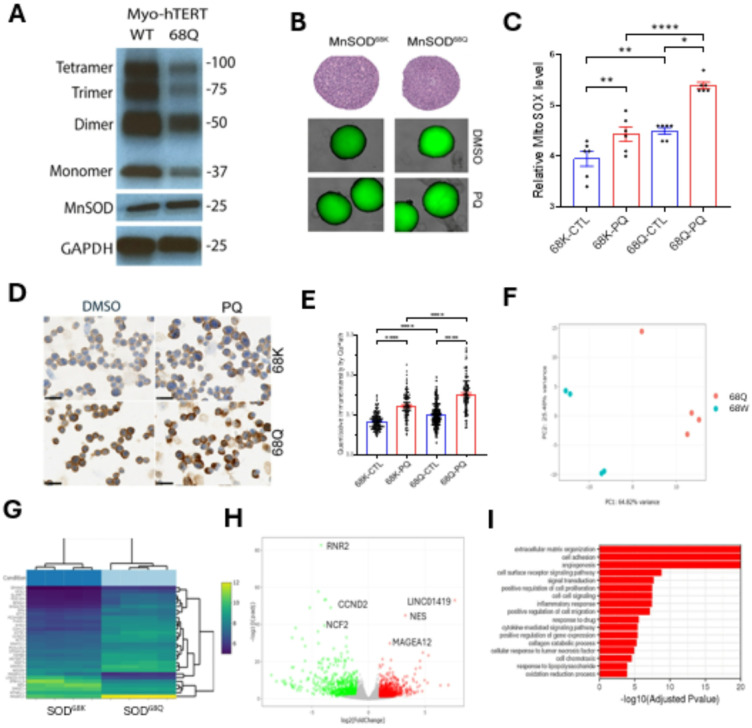
Cellular and molecular characteristics of myo-hTERT cell lines with MnSOD 68K to 68Q site mutants in vitro. **A.** Western blot demonstrated the reduction of tetrameric and trimeric MnSOD species in MnSOD^68Q^ cell line in comparison to MnSOD^68K^. Total MnSOD and GAPDH loading controls were provided. **B.** Spheroids of MnSO^68K^ and MnSO^68Q^ myo-hTERT cell lines showed different ROS productions with and without PQ treatment detected by DHE stain. **C.** Dot plot illustrated mitochondrial superoxide accumulation detected by MitoSOX stains in MnSOD^68Q^ than MnSOD^68K^ with and without PQ treatment. **D-E.** Immunohistochemistry (**D**) and quantitative analysis (**E**) of HO1 expression in MnSOD^68Q^ than MnSOD^68K^ with and without PQ treatment. **F.** PCA analysis of RNA expression profile revealed distinct clusters of MnSOD^68K^ (blue) and MnSOD^68Q^ (red) cells without PQ treatment. **G-H.** Heatmap (**G**) and volcano plot (**H**) demonstrated differentially expressed genes in myo-hTERT cells with MnSOD^68K^ and MnSOD^68Q^ mutants. **I.** KEGG analysis^18–20^ highlighted the top-ranking functional pathways in myo-hTERT with MnSO^68Q^. **J-K.** Heatmap and KEGG analysis showed the differen26tially expressed genes (**J**) and top-ranking pathways (**K**) in MnSOD^68Q^ myo-hTERT with and without PQ treatment. *: p<0.05; **: p<0.01; ***: p<0.001; ****: p<0.0001.

In this study, we established Myo-hTERT cell lines with stable overexpression of MnSOD with lysine at K68 (MnSOD^68K^) and site mutants of glutamine (MnSOD^68Q^, acetylation mimetic) by lentiviral constructs (see Methods, **Suppl Fig. S1A**)^12^. Western blot analysis confirmed the reduction of tetrameric and trimeric MnSOD species in MnSOD^68Q^ cell lines in comparison to MnSOD^68K^, consistent with previous reports describing structural changes associated with acetylation at lysine 68^9^ (Fig. 1A). To establish conditions for oxidative stress induction, myo-hTERT cell lines were treated with increasing concentrations of PQ; 100 μM produced a robust oxidative stress response without excessive toxicity and was therefore selected for all experiments. (**Suppl Fig. S1B**). Cells were cultured as 3D spheroids (Fig. 1B) to better approximate ex vivo tissue architecture.

Following PQ treatment, both MnSOD^68K^ and MnSOD^68Q^ Myo-hTERT cell lines exhibited increased ROS production as measured by DHE staining. and MnSOD^68Q^ cells displayed elevated ROS levels even in the absence of PQ compared with MnSOD^68K^, suggesting impaired oxidative clearance in the acetylation-mimetic genotype (Fig. 1B). This was demonstrated by a significantly higher mitochondrial superoxide accumulation detected by MitoSOX stains in MnSOD^68Q^ than MnSOD^68K^ (Fig. 1C). Markers of oxidative stress and DNA damage were also induced by PQ treatment, including HO1, a sensitive regulator of ROS-responsive pathways (Fig. 1D and 1E), H2AX (DNA damage) and 8OHdG (Oxidized Guanine) (**Suppl Fig. S1C**). Baseline levels of these markers were elevated in MnSOD^68Q^ cells compared with MnSOD^68K^ cells under vehicle conditions, further supporting that acetylation-mimetic MnSOD compromises antioxidant defense. Taken together, these finds demonstrate that acetylation-dependent modification of MnSOD at lysine 68 impairs its antioxidant capacity, leading to increased mitochondrial ROS accumulation and altered stress-response signaling in myometrial cells under oxidative stress conditions.

### Transcriptomic profiling of myometrial cells expressing the MnSOD-K68 acetylation mimic

To evaluate the global gene expression changes associated with MnSOD acetylation at lysine 68, a global transcriptomic analysis was performed in Myo-hTERT cell lines expressing either wild-type MnSOD^68K^ or the acetylation-mimetic mutant MnSOD^68Q^. Four to five clones from each genotype were maintained in medium with PQ treatment or vehicle control and subjected to transcriptomic analysis. A PCA analysis of the global RNA expression profile revealed a clear separation of MnSO^68K^ and MnSO^68Q^ cells without PQ treatment (Fig. 1F) and a total of 1,588 differentially expressed genes (DEGs) with FDR < 0.05, |log_2_FC| >1 was identified (Fig. 1G and 1H, **Supple Table S1**). DEGs included 869 upregulated and 719 down regulated and top ranking DEGs were highly relevant to MnSOD, and mitochondrial functions highlighted in Fig. 1H. Pathway analysis demonstrated that MnSOD^68Q^ dominated ECM, cell adhesion, inflammation, oxidative stress, angiogenesis, and cell proliferation (Fig. 1I). Similar gene expressions, profiles, and pathways were observed in these two cell lines with PQ treatment (**Suppl Fig. S2A-2C**). Transcriptomic analysis demonstrates that myometrial cell lines with MnSOD^68Q^ greatly impact the global gene expression involving many functional pathways and cellular processes.

### Establish chronic oxidative stress in myometrial xenografts in vivo

**Figure 2:**
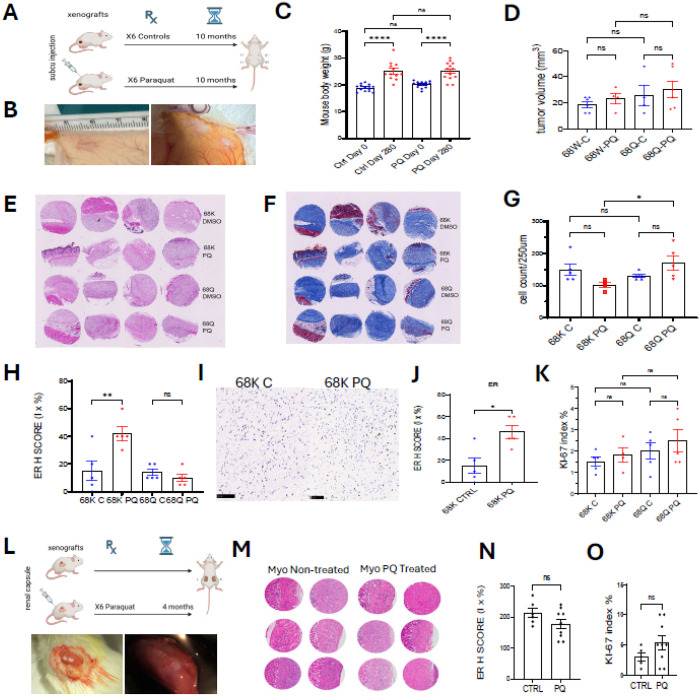
Cellular and phenotypical analysis of primary myometrial and MnSOD-deficient cell line xenografts with chronic oxidative exposure in vivo. **A.** Diagram illustrated subcutaneous xenografts of MnSOD^68K^ and MnSOD^68Q^ myo-hTERT cell lines with PQ treatment up to 40 weeks. **B.** Photomacrographs of subcutaneous xenografts for myo-hTERT cell line tumor nodules. **C.** The mouse body weights (g) with and without PQ treatment at harvest time. **D.** The tumor xenograft volume (mm^3^) at harvest time. **E-F.** Photomicrographs of H/E stained (**E**) and trichrome stained (**F**) tissue microarray sections from MnSOD^68K^ and MnSOD^68Q^ myo-hTERT cell xenografts with and without PQ. **G.** The cell counts in MnSOD^68K^ and MnSOD^68Q^ myo-hTERT cell xenografts with and without PQ. **H-J.** Estrogen receptor (ER) expression was increased in MnSOD^68K^ myo-hTERT cell line in vivo (**H, I**) and in vitro (**J**). **K.** Cell proliferation index detected by Ki-67 immunohistochemistry in different cell types and treatment. **L.** Diagram (upper) and Photomacrographs (lower) illustrated primary myometrial xenografts at renal capsule. **M.** Photomicrograph of tissue microarray sections from xenografts of primary myometrial nodules. **N-O.** ER expression (N) and Ki-67 index in myometrial xenografts by immunohistochemistry analysis. ns: no significance; *: p<0.05; **: p<0.01; ***: p<0.001; ****: p<0.0001.

To investigate the impact of chronic oxidative stress on human myometrial cells with either wild-type MnSOD (MnSOD^68K^) or the acetylation-mimetic mutant (MnSOD^68Q^) in an *in vivo* setting, Myo-hTERT cells (5 × 10^5^) suspended in Matrigel were injected subcutaneously into NOD mice (Fig. 2A). Mice were then provided drinking water either without (control group n = 6) or with PQ (test group, n = 6) to induce chronic oxidative stress (see Methods) in accordance with approved animal care protocols. Myo-hTERT xenografts were harvested at 40 weeks (Fig. 2B). Mice gained up to 30% body weight in both control and test groups, indicating no significant toxicity of PQ (Fig. 2C). The tumor volume with PQ treatment was slightly larger than control group but did not reach statistical significance (p > 0.05, Fig. 2D).

The histology and cellular organization in myometrial cell lines were reviewed by pathologists (**Suppl Fig. S3A**) and tissue microarrays (TMA) from formalin-fixed and paraffin-embedded (FFPE) tissues were prepared (Fig. 2E). There were no differences in nuclear size, chromatin, cellular arrangement, and nuclear/cytoplasmic ratio between control and test groups, or different MnSOD genotypes (Fig. 2E). The trichrome stain highlighted the presence of evenly distributed but focally concentrated collagen, indicating the organized extracellular matrix (Fig. 2F). The cellularity tended to be slightly higher in MnSOD^68Q^ with PQ treatment compared to no treatment (Fig. 2G). Estrogen receptor (ER) was significantly induced in MnSOD^68K^, but not in MnSOD^68Q^ (Fig. 2H and 2I). PQ-induced ER expression was also observed in MnSOD^68K^ myo-hTERT spheroids in vitro (Fig. 2J). The cell proliferation index detected by Ki-67 tended to be slightly increased in MnSOD^68Q^ with PQ treatment but did not reach statistical significance (Fig. 2K).

Human primary myometrial cells were also evaluated under conditions of chronic oxidative stress *in vivo*. Cell pellets of primary myometrial cells embedded in Matrigel were xenografted beneath the renal capsule of mice (Fig. 2L). To control the hormonal environment, recipient mice were ovariectomized and supplemented with subcutaneous estradiol (E2) and progesterone (P4) pellets. Primary myometrial xenografts (PDX) were harvested after 16 weeks. Formalin-fixed and paraffin-embedded myometrial nodules were examined histologically and revealed normal appearing myometrial smooth muscle cells with preserved growth architecture. (**Suppl Fig. S3B**, Fig. 2M). ER expression was slightly reduced in myometrial PDX nodules with PQ treatment (Fig. 2N). No significant difference of Ki-67 index was observed (Fig. 2O). Together, these findings demonstrate that chronic PQ-induced oxidative stress can be established in myometrial xenografts without overt toxicity, producing modest cellular and molecular alterations, particularly in extracellular matrix organization and estrogen receptor signaling while largely preserving normal myometrial histology and proliferative capacity.

### Spatial transcriptomic analysis of MnSOD ^68K^ xenografts under chronic oxidative stress

**Figure 3:**
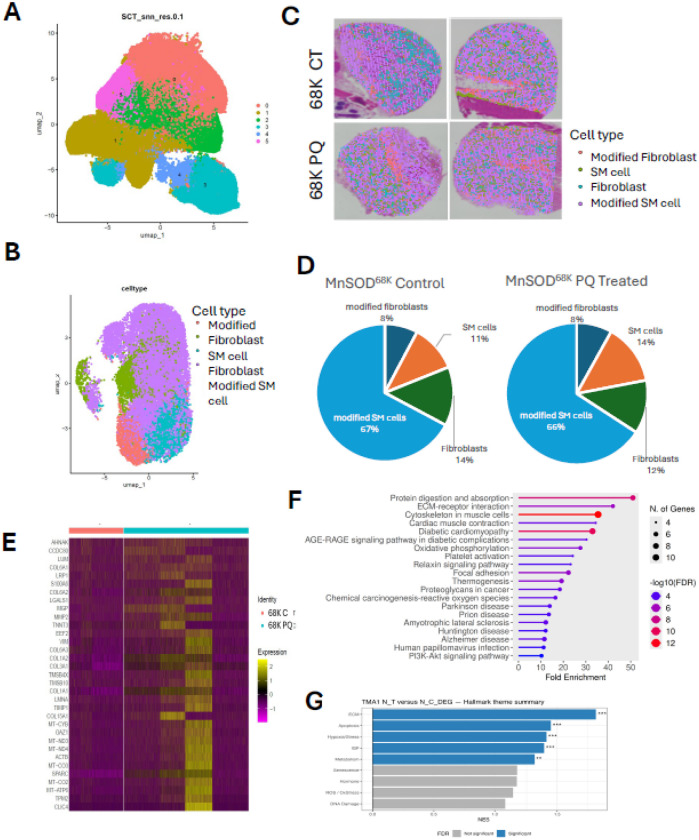
Spatial transcriptomic analysis of MnSOD68K xenografts under chronic oxidative stress. A. RNA signature of 0–5 mapped in different colors were generated from myo-hTERT xenografts. B. UMAP illustrated the graphic clusters of 4 cell types detected by cell specific RNA signature from myo-hTERT xenografts. C. A spatial transcriptomic map showed the distribution of the dominant cell types in MnSO68K cell xenografts with and without PQ treatment. D. The color pier showed the percentage of each of 4cell types between control and PQ treatment. E. Heatmap illustrated the top ranking differentially expressed genes in MnSOD68K cell xenografts with and without PQ treatment. F. GO pathway analysis demonstrated the top-ranking pathways in PQ treated MnSOD68K cell xenografts (ShinyGO 0.85 https://bioinformatics.sdstate.edu/go/). G. The hallmark theme summary analysis highlighted ROS mediated pathways that were significantly activated in MnSOD68K cell with PQ treatment.

Myo-hTERT xenografts exhibited slow subcutaneous growth, and chronic paraquat (PQ) exposure was therefore maintained for 40 weeks before termination of the experiment (Fig. 2). To characterize global gene expression and spatial transcriptional organization within the xenografts, a high-density single cell spatial transcriptomic analysis was performed. Using the TMA, up to 5 xenograft nodules from each of four conditions (MnSOD^68K^, and MnSOD^68Q^ myo-h-TERT nodules with and without PQ treatment (Fig. 2E) were arrayed and subjected to high density transcriptomic analysis (See Methods). A total of 6 distinct RNA signatures were identified in Myo-hTERT cells (Fig. 3A). Cell type specific RNA signatures identified 4 major cell types, including smooth muscle, modified smooth muscle, fibroblast, and modified fibroblast cell types (Fig. 3B) and gene signatures for each cluster were illustrated in **Suppl Table S2**. The spatial and regional distribution of these 4 cell types on MnSOD^68K^ myometrial cell nodules of with and without PQ treatment are shown in Fig. 3C. The data demonstrated the cell lines maintained low heterogeneity after long-term in vivo growth. The dominant cell population was cells with gene signatures, associated with cellular stress-response in transcriptional adaptation, consistent with a remodeling smooth muscle cell state for altered cytoskeletal and contractile structure include DES, TNNI1, PFN1, TNS2, and NES, this is consistent with modified smooth muscle cells under long term xenograft. The other three cell types showed gene signatures defined as smooth muscle, fibroblasts and modified fibroblasts, and top-ranking genes were summarized in **Suppl Fig S4A-4D and Suppl Table S2**. The modified fibroblasts reflected a differentiation state through activation of stromal fibroblast/myofibroblast-like population characterized by strong extracellular matrix (ECM) production and remodeling. There were no significant differences in the proportions of 4 cell types between control and PQ treatment, indicating chronic oxidative stress may not promote further myometrial cell differentiation (Fig. 3D).

Next, the differential expression genes (DEGs) of wild type MnSOD^68K^ myo-hTERT with or without chronic PQ treatment were analyzed. A total of 192 DEGs with FDR < 0.05, |log_2_FC| >1, included 38 upregulated and 154 down regulated (**Suppl Table S3**). The top ranking DEGs are summarized in Fig. 3E. Notably, the major pathways induced by chronic oxidative stress in MnSOD^68K^ cells include ECM, ROS, muscle cytoskeleton, PI3K-AKT pathways (Fig. 3F). This is consistent with our in vitro findings (Fig. 2) and previously published data^4^(see discussion). Importantly, the hallmark theme summary demonstrated significant activation of ECM, apoptosis, hypoxia and IGF pathways (Fig. 3G). This highlights the key molecular changes commonly seen in uterine LMs.

### Spatial transcriptomic analysis of MnSOD ^68Q^ xenografts under chronic oxidative stress

**Figure 4:**
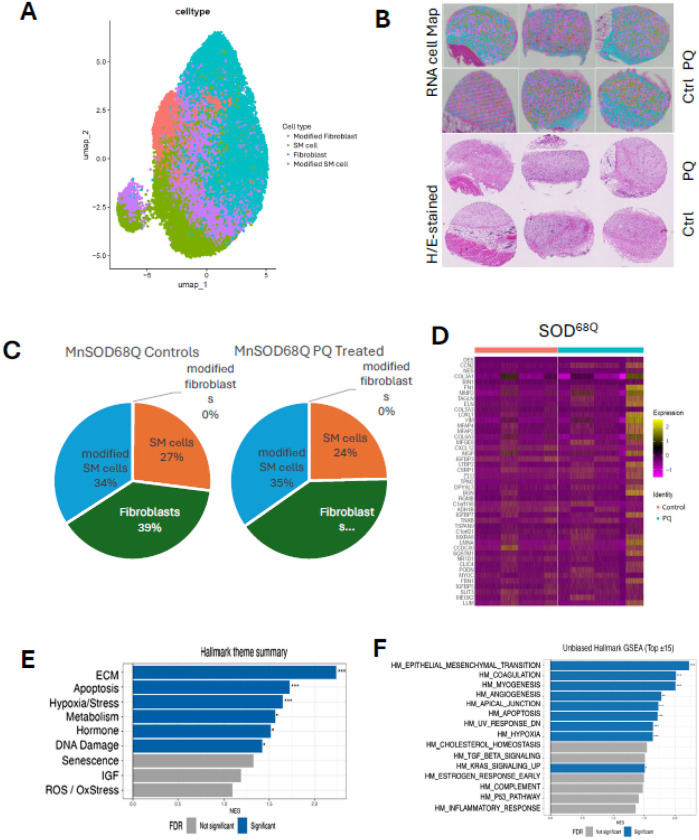
Spatial transcriptomic analysis of MnSOD^68Q^ xenografts under chronic oxidative stress. **A.** UMAP illustrated the graphic clusters of 4 cell types detected by cell specific RNA signature from MnSOD^68Q^ myo-hTERT xenografts. **B.** A spatial transcriptomic map showed the distribution of the dominant cell types in MnSOD^68Q^ cell xenografts with and without PQ treatment. **C.** The color pier showed the percentage of each of 4 cell types between control and PQ treatment. **D.** Heatmap illustrated the top ranking differentially expressed genes in MnSOD^68Q^ cell xenografts with and without PQ treatment**. E-F.** The hallmark theme summary (**E**) and Unbiased Hallmark GSEA (**F**) analysis highlighted ROS mediated pathways that were significantly activated in MnSOD^68Q^ cell with PQ treatment.

Similar to MnSOD^68K^ myometrial cell xenografts, under chronic PQ treatment, MnSOD^68Q^ myometrial cells differentiated into 4 major cell types based on cell type specific RNA signatures, including smooth muscle, modified smooth muscle, fibroblast, and modified fibroblast cell types (Fig. 4A). The spatial and regional distribution of these 4 cell types on MnSOD^68Q^ xenografts with and without PQ treatment are shown in Fig. 4B. The dominant cell population in MnSOD^68Q^ myometrial cells were fibroblasts, followed by smooth muscle and modified smooth muscle cells (Fig. 4C). A very small fraction of modified fibroblasts was detected. There were no significant differences in the ratio of 4 cell types between control and PQ treatment (Fig. 4C). DEGs of MnSOD^68Q^ myo-hTERT with and without chronic PQ treatment were analyzed. A total of 388 DEGs with FDR < 0.05, |log_2_FC| >1, included 211 upregulated and 177 down regulated (**Suppl Table S4**). The top ranking DEGs are summarized in Fig. 4D.

We then compared the top-ranking curated hallmark pathways for PQ treated MnSOD^68Q^ to the non-PQ-treated MnSOD^68Q^ and found a significant upregulation of ECM, apoptosis, metabolism, hormone, DNA damage pathways in the PQ treated MnSOD^68Q^ xenograft (Fig. 4E). Furthermore, an unbiased Hallmark pathway analysis of these two groups showed upregulation of EMT, myogenesis, angiogenesis, and Kras signaling pathways (Fig. 4F), indicative of high risk of tumorigenesis in MnSOD^68Q^ cells with chronic ROS exposure.

### Spatial transcriptional analysis of primary myometrial cells under chronic oxidative stress

**Figure 5:**
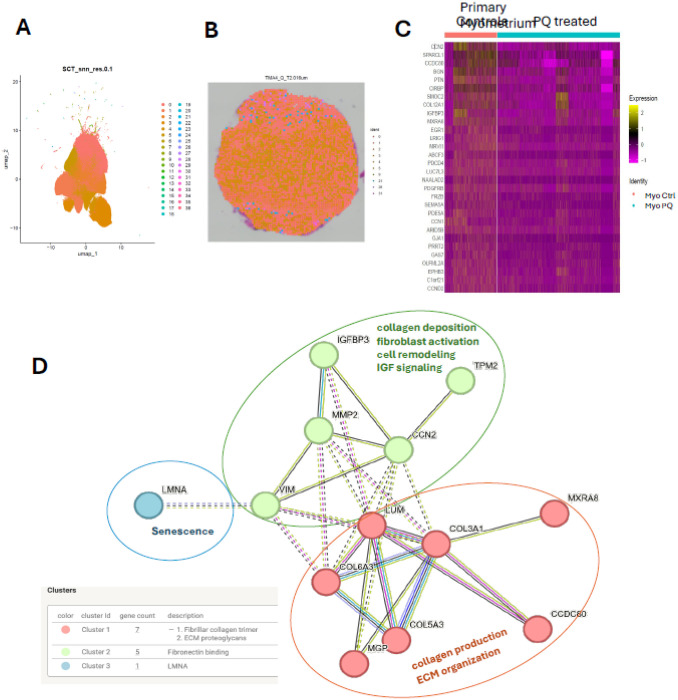
Spatial transcriptional analysis of primary myometrial cells under chronic oxidative stress. **A.** RNA signature of 0–36 mapped in different colors were generated from primary myometrial cell xenografts. **B.** An example of spatial transcriptomic map showed the distribution of the dominant cell types in myometrial cell xenografts with PQ treatment. **C.** Heatmap illustrated the top ranking differentially expressed genes in MnSO^68Q^ cell xenografts with and without PQ treatment**. D.** Cluster analysis of functional connection in 13 significant dysregulated genes in all three cell types (MnSO^68K^ and MnSO^68Q^ and primary myometrial cells) with PQ treatment.

Primary myometrial cells are very difficult to grow in NOD mice. Previously, we established patient derived xenografts (PDX) model of myometrial cells in renal capsule with success^21^. In this study, we collected primary myometrial cells from hysterectomy specimens. PDX was established in 8-week NOD mice with oophorectomy followed by E2/P4 supplement subcutaneous pellet (Fig. 2K and 2L). Chronic PQ treatment was given up to 16 weeks. Myometrial nodules were collected and evaluated by histology (**Suppl Fig. S3B**) and spatial transcriptomic RNA sequencing was performed. Transcriptomic analysis revealed 37 gene signatures with 10 dominant clusters of smooth muscle, fibroblast, stromal/mesenchymal, and inflammatory cells (Fig. 5A and 5B). The DEGs of primary myometrial cells with and without chronic PQ treatment were analyzed and 1714 DEGs with FDR < 0.05, |log_2_FC| >1, included 73 upregulated and 1641 down regulated (**Suppl Table S5**). The 30 top ranking DEGs are summarized in Fig. 5C. A downregulation of most DEGs in PQ-treated primary myometrial cells is likely contributed to their sensitivity to stress in grafting environment than immortalized cells due to their in vivo-like characteristics and complex signaling. Primary myometrial cells mimic healthy tissue better but struggle with limited lifespan and specific growth needs, making them highly responsive to many stressors.

We then compared the DEGs in three different myometrial cell types (xenografts of Myo-hTERT MnSOD^68K^ and MnSOD^68Q^, and primary myometrial cells) with and without PQ treatment. Among top 50 DEG from three cell types with PQ treatment, 26% (13/50) of DEG genes were shared. Analysis by functional protein association networks (STRING-DB.ORG) demonstrated three functionally connected clusters highly relevant to extracellular organization, cell remodeling, and fibroblast activation (Fig. 5D). The findings suggest that long-term and low dose of chronic oxidative exposure in vivo can enhance collagen production, ECM remodel, hallmarks of leiomyomagenesis.

### MED12 mutation analysis in primary myometrial xenografts under chronic oxidative stress

**Figure 6:**
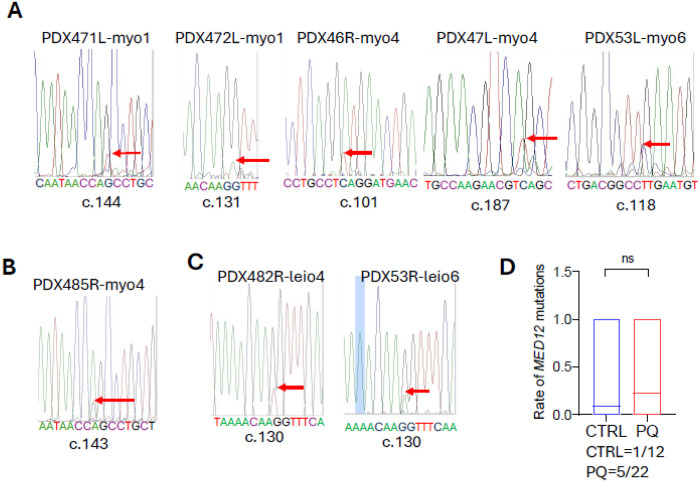
MED12 mutation analysis in primary myometrial xenografts under chronic oxidative stress. **A.** *MED12* exon2 mutations (red arrows) in five myometrial xenografts with PQ treatment. **B.** *MED12* exon2 mutations (red arrow) in one myometrial xenograft without PQ treatment. **C.** *MED12*exon2 mutations (red arrows) in two leiomyoma xenografts with known c.130 mutation. **D.** The rate of *MED12*exon2 mutations in myometrial xenografts with and without PQ treatment.

We previously demonstrated that chronic PQ treatment of myometrial cells promoted mutations in *MED12* exon 2^4^. Thus, we examined whether chronic oxidative stress promotes the emergence of *MED12* mutations in primary myometrial xenografts. Mutation analysis of *MED12* exon2 was done by Sanger sequencing the primary myometrial xenografts. Mutations were considered present when a specific point mutation was detected in more than 10% of sequenced cells. Myometrium was collected from 6 patients and multiple xenografts were generated from each specimen. All primary myometrial samples were sequenced prior to xenografting, and no *MED12* mutations were detected. Two leiomyoma xenografts harboring known *MED12* c.130 mutations were included as positive controls. As shown in Fig. 6, one mutation was detected in a nontreated group, accounting for 8.33% (1/12) cases. In contrast, 22.7% (5/22) myometrium with PQ treatment harbored *MED12* mutations in different sites of exon 2. Two leiomyomas with c.130 mutations were xenografted as positive controls (Fig. 6C). Although the increased mutation frequency in the PQ-treated group did not reach statistical significance due to the limited sample size (Fig. 6D), the higher mutation rate observed under oxidative stress conditions suggests that chronic oxidative stress may promote DNA misrepair and the acquisition of *MED12* mutations. Together, these results suggest that chronic oxidative stress may increase the frequency of *MED12* mutations in myometrial cells in an in vivo setting implicating oxidative DNA damage as a potential early driver of leiomyoma development.

## DISCUSSION

Previously, we demonstrated that uterine leiomyoma and adjacent myometrium exist in a highly oxidative environment characterized by increased MnSOD acetylation, reduced MnSOD catalytic activity, and elevated ROS levels^4,9,24^, suggesting that oxidative stress may represent a permissive environment for tumor initiation. However, the mechanistic link between chronic oxidative stress and early leiomyoma development has remained unclear as these observations were correlative and did not establish whether impaired MnSOD function directly contributes to oxidative stress–driven cellular and genomic changes. Our findings show that sustained oxidative stress in myometrial cells using the engineered acetylation mimic MnSOD myometrial cell lines or long-term treatment with PQ, induced transcriptional changes and extracellular matrix remodeling similar to those seen in leiomyomas, and could also promote genomic alterations in the *MED12* gene.

Increasing oxidative stress in subcutaneous xenograft models allows investigators to determine whether redox mechanisms observed in vitro influence cell behavior in a more physiologically relevant setting. Unlike cell culture systems, xenografts expose human cells to a living tissue environment that includes extracellular matrix interactions, vascularization, and host-derived stromal components that can influence oxidative stress responses. Importantly, these models permit long-term experiments that allow sustained oxidative stress to be imposed over extended periods, enabling the study of cellular adaptation to chronic ROS exposure. This prolonged in vivo context makes it possible to evaluate integrated outcomes such as changes in growth, tissue organization, and long-term cellular responses that cannot be captured in short-term in vitro experiments. We also xenografted cells derived from primary human myometrium under the kidney capsule, a site characterized by greater vascularization^16^. In this model, PQ exposure for 16 weeks resulted in DEGs of 1,714 genes, and alterations of functional programs including pathways related to metabolism, hormone signaling, apoptosis, hypoxia, and extracellular matrix remodeling. Although PQ was administered orally at a relatively low systemic dose via drinking water for 16 weeks, a steady kidney uptake of PQ is usually higher than other organs in the body^25^ and thus PQ is able to reach the graft site efficiently, given the high vascularity of the site. The combination of PQ exposure and the fragility of the myometrial graft may have impacted the integrity of the tissue as some grafts exhibited degenerative change.

Cell cluster analysis in the Myo-hTERT cell line models, present in both the MnSOD^−68K^ and MnSOD^−68Q^ conditions, showed smooth muscle cell and fibroblast differentiation. One of the cell clusters identified was modified fibroblastic-like cell population. This designation reflects a differentiation state resembling these cells with activated form for strong extracellular matrix production and remodeling, suggesting that a subset of myometrial cells adopts a more differentiated phenotype in response long-term oxidative exposure. Gene expression analysis of this cluster revealed that modified cells gain ECM synthesis and organization, matrix remodeling and processing, and profibrotic signaling. The presence of modified cell clusters in both conditions indicates that myometrial cells retain the capacity to undergo stress-mediated differentiation despite alterations in redox balance. In the spatial transcriptomic analysis, one cluster of cells was annotated as “modified smooth muscle cell” because its transcriptional profile did not clearly match established marker signatures for the major cell populations expected in the grafts, such as smooth muscle, fibroblast, stromal/mesenchymal, or inflammatory cells. Gene expression within this cluster demonstrates three related biological programs, including contractile organization, ECM remodeling and stress-response with transcriptional adaptation. The gene signature of this cluster is more consistent with remodeling or activating smooth muscle cell differentiation when cells are under chronic oxidative conditions. The presence of this cluster highlights the cellular heterogeneity within the grafts and suggests that prolonged oxidative stress may generate cell states change so that they are not easily assigned to canonical myometrial lineages.

*MED12* mutations detected in this study did not consistently converge on the canonical hotspot at c.130, and the overall number of mutations observed was limited by the relatively small sample size. This variability is not unexpected, as our previous studies demonstrated that *MED12* mutations induced by chronic paraquat exposure arise in only a small subset of cells. Future studies using larger cohorts and longer observation periods will be necessary to confirm the role of oxidative stress in MED12 mutagenesis and leiomyoma initiation. Despite these limitations, mutations in the MED12 gene were observed following prolonged oxidative stress, supporting the concept that chronic ROS exposure can promote genomic alterations in myometrial cells that are relevant to leiomyoma initiation.

In summary, our findings provide mechanistic evidence that sustained oxidative stress alters myometrial cell behavior and promotes molecular changes associated with leiomyoma development. By inducing chronic redox imbalance through impaired MnSOD function and prolonged oxidative exposure, we show that myometrial cells undergo transcriptional and phenotypic adaptations resembling pathways observed in leiomyomas. These results suggest that long-term oxidative stress may contribute to leiomyoma initiation and highlight mitochondrial redox regulation as an important determinant of myometrial adaptation to chronic stress. Understanding these mechanisms may ultimately inform the development of therapeutic strategies aimed at restoring redox balance or targeting oxidative stress pathways to prevent or slow leiomyoma formation.

## Supplementary Material

Supplementary Files

This is a list of supplementary files associated with this preprint. Click to download.

- SupplTablesfinal3.26.xlsx
- SupplFiguresrevised5.2.26.pdf

## Data Availability

The datasets generated and/or analyzed during the current study are available in the GEO re-pository with accession number: GEO330163
